# An unsupervised partition method based on association delineated revised mutual information

**DOI:** 10.1186/1471-2105-10-S1-S63

**Published:** 2009-01-30

**Authors:** Jing Chen, Guangcheng Xi

**Affiliations:** 1 Key Laboratory of Complex Systems and Intelligence Science, Institute of Automation, Chinese Academy of Sciences, Beijing, 086-100190, PR China

## Abstract

**Background:**

The syndrome is the basic pathological unit and the key concept in traditional Chinese medicine (TCM) and the herbal remedy is prescribed according to the syndrome a patient catches. Nevertheless, few studies are dedicated to investigate the number of syndromes and what these syndromes are. Correlative measure based on mutual information can measure arbitrary statistical dependences between discrete and continuous variables.

**Results:**

We presented a revised version of mutual information to discriminate positive and negative association. The entropy partition method self-organizedly discovers the effective patterns in patient data and rat data. The super-additivity of cluster by mutual information is proved and N-class association concept is introduced in our model to reduce computational complexity. Validation of the algorithm is performed by using the patient data and its diagnostic data. The partition results of patient data indicate that the algorithm achieves a high sensitivity with 96.48% and each classified pattern is of clinical significance. The partition results of rat data show the inherent relationship between vascular endothelial function related parameters and neuro-endocrine-immune (NEI) network related parameters.

**Conclusion:**

Therefore, we conclude that the algorithm provides an excellent solution to patients and rats data problem in the context of traditional Chinese medicine.

## Background

Traditional Chinese medicine (TCM) is taken by most people in China as a complementary therapeutic alternative since herbal remedies have the advantage over western medicine in that it has less side effects and are less costly. TCM has been always regarded as a key component in 5000 years of Chinese civilization history. In ancient times before modern medicine was born, people all over the world mainly benefit from three traditional medicines, among which only TCM is still alive today; while Chaldaic and ancient Hindu medicines only have extremely rare documents as evidence that they ever existed in history. TCM, whose core is syndrome, is on the way to modernization. It is aiming to be accepted, like Western medicine, as a science [1-3].

The syndrome is the basic pathological unit and the key concept in TCM theory since herbal remedy is prescribed according to syndrome or syndromes a patient catches [4]. Therefore, identification and determination of syndrome(s) in TCM become significantly important for TCM physicians. Nevertheless, there are few documents dedicated to this issue.

In information theory, entropy is a metric to measure uncertainty of random variables. Mutual information (MI) of two random variables is a measure that scales mutual dependence of the two variables. It has been applied in many fields, in which researchers treat as divergence or distance between two distributions [5-7]. The advantage of mutual information over correlation methods is discussed in [8]. In this paper, we propose a novel unsupervised data mining model, in which we treat mutual information as an association measure of two variables. In our effort, we try to unsupervisedly discover syndromes in chronic renal failure (CRF) data and clinically verify these syndromes to test the performance of our model. Based on revised mutual information, we propose an unsupervised pattern discovery algorithm to self-organizedly allocate significantly associated symptoms to patterns. By using diagnostic patients data, each pattern is verified to have clinical meaning. By using rats data, we also apply this method to find the inherent relationship between vascular endothelial function related parameters and NEI network related parameters.

## Methods

### Correlative measure based on mutual information

#### Correlative measure for discrete variables

Mutual information between two discrete variables is formally defined as:

(1) *MI*(*X*, *Y*) = *H*(*X*) + *H*(*Y*) - *H*(*X *∪ *Y*)

where *H*(*X*) denotes the Shannon entropy of variable *X*, *H*(*X *∪ *Y*) represents the joint entropy between variables *X *and *Y*. Formally, suppose that *X *and *Y *are both categorical variables, *H*(*X*) and *H*(*X *∪ *Y*) are denoted as:

(2) $$H\left(X\right)=-\sum_{i=1}^{m}\frac{n_{i}}{N}\ln\frac{n_{i}}{N}$$

where *n*_*i *_denotes the number of occurrence of the *i*th category of *X *with *m *categories, *N *is the total number of sampled *X*

(3) $$H\left(X\cup Y\right)=-\sum_{i=1}^{m}\sum_{j=1}^{l}\frac{n_{ij}}{N}\ln\frac{n_{ij}}{N}$$

where *n*_*ij *_represents the number of simultaneous occurrence of the *i*th category of *X *with *m *categories and the *j*th counterpart of *Y *with *l *categories.

Mutual information is universally used to measure the similarity between two variables' distributions and is taken here as an association measure of two variables. Indeed, MI is a measure defined on the set consisted of two variables, if the set is composed of more than two variables, then the definition of MI measure will be rewritten as follows:

(4) $$MI\left(X_{1},X_{2},\ldots X_{n}\right)≜\sum_{i=1}^{n}H\left(X_{i}\right)-H\left(\overset{n}{\underset{i=1}{\cup }}X_{i}\right)$$

Mutual information has an interesting property – super-additivity. We introduce the concept of super-additivity and give the mathematical proof of it. However, it is noted that the super-additivity of mutual information has minor contribution to validation of the algorithm here. Text for this section.

#### Super-additivity of correlative measure

Let us consider nonempty finite set *X *and set-family *E*(*X*) consisting of its subsets *P *is a set-function defined on *E*(*X*) with properties:

(i) *P*(*A*) ≥ 0, ∀ *A *∈ *E*(*X*)

(ii) *P*(∅) = 0

If for arbitrary nonempty finite set *S*_*i *_∈ *E*(*X*), *S*_*j *_∈ *E*(*X*), *i *≠ *j*, *S*_*i *_∈ *S*_*j *_= *ϕ*, have

(5) *P*(*S*_*i *_∪ *S*_*j*_) ≥ *P*(*S*_*i*_) + *P*(*S*_*j*_)

This set-function *P *is called super-additive.

One of the important properties of correlative measure is just its super-additivity. In other words, correlative measure of one finite set is no less than the summation of the correlative measures of all its subsets.

**Theorem**. Correlative measure *MI*(*s*_1_, *s*_2_, ⋯, *s*_*m*_) is finitely super-additive, and unique.

**Proof**. The definition of the MI ensures the uniqueness of the measure. We now turn to prove super-additivity of the measure. Suppose that the set *X ψ *is partitioned into *m ψ *subsets *s*_1_, *s*_2_,⋯, *s*_*m *_satisfying for arbitrary *i*, *j *(*i *≠ *j*), *s*_*i *_≠ 0, *s*_*j *_≠ 0,

$$X=\cup_{i=1}^{m}s_{i}=\cup_{s_{i}\in X}s_{i}$$

We only need to prove

(6) $$MI(\overset{m}{\underset{i=1}{\cup }}s_{i})\geq \sum_{i=1}^{m}MI(s_{i})$$

By the definition of MI, we have

(7) $$\begin{array}{c} MI(X)=MI(\overset{m}{\underset{i=1}{\cup }}s_{i})=MI(s_{1},s_{2},...s_{m})\\ =MI(X_{1},X_{2},...X_{n})\\ =\sum_{i=1}^{n}H(X_{i})-H\left(\overset{n}{\underset{i=1}{\cup }}X_{i}\right) \end{array}$$

(8) $$\begin{array}{c} \sum_{s_{i}\in X}MI(s_{i})=\sum_{s_{i}\in X}\left(\sum_{X_{j}\in s_{i}}H(X_{j})-H(\sum_{X_{j}\in s_{i}}X_{j})\right)\\ =\sum_{i=1}^{n}H(X_{i})-\sum_{s_{i}\in X}H(s_{i})\\ =\sum_{i=1}^{n}H(X_{i})-\sum_{i=1}^{m}H(s_{i}) \end{array}$$

Subtracting (7) from (8), we have

(9) $$\begin{array}{c} MI(X)-\sum_{s_{i}\in s}MI(s_{i})=\sum_{i=1}^{m}H(s_{i})-H\left(\overset{n}{\underset{i=1}{\cup }}X_{i}\right)\\ =\sum_{i=1}^{m}H(s_{i})-H(\left(\overset{m}{\underset{i=1}{\cup }}s_{i}\right))\geq 0 \end{array}$$

The proof is complete.

#### A revised version of correlative measure

Despite so many merits of applying MI have been recorded [9], MI also suffers from some defects when dealing with the data. First, MI-based association between two variables is symmetric, but the relation between two symptoms is usually asymmetric. Indeed, symmetric is a special case of asymmetric. Alternatively, two variables' MI is non-negative but boundless, which may make evaluating two subjects' relation difficult in a situation that the association value is isolated. An ameliorated version of the MI can fill the gap. We used the normalized form of association between two variables *μ *as:

(10) $$\mu (X,Y)=\frac{MI(X,Y)}{H(Y)}$$

By this definition, the relation between two variables is asymmetric because two variables' Shannon entropies are usually difficult. Additionally, according to information theory, *MI*(*X*, *Y*) is non negative and its upper bound is the minimum between *H*(*X*) and *H*(*Y*), therefore, the new version of association *μ*(*X*, *Y*) takes value between 0 and 1, which is similar to correlation in statistical theory to some extent.

Furthermore, by information theory, the form of MI can be recast as:

(11) *MI*(*X*, *Y*) = *H*(*X*) - *H*(*X*|*Y*)

where *H*(*X*|*Y*) denotes conditional entropy, it measures the remaining uncertainty of *X *under the condition of knowing *Y*, that is to say, *MI*(*X*, *Y*) represents the information content with regard to knowing *X *under the condition of knowing *Y*. Therefore, associations of two mostly close symptoms and completely opposite counterpart are both very large, making the association defined by MI compose of positive association and negative one. We present an ameliorated version of MI to distinguish positive association and negative association.

The frequency that *X *and *Y *are both of nonzero categories is denoted as *Pofr*(*X*, *Y*), it is this positive frequency of *X *and *Y *that separates positive association and negative association. We redefined the form of MI as:

$$MI\left(X,Y\right)=\{\begin{array}{l} \frac{H\left(X\right)+H\left(Y\right)-H\left(X\cup Y\right)}{H\left(Y\right)},pofr\left(X,Y\right)\geq \theta \\ \frac{H\left(X\right)+H\left(Y\right)-b*H\left(X\cup Y\right)}{H\left(Y\right)},pofr\left(X,Y\right)<\theta \end{array}$$

where *θ *is pre-assigned positive quantity, we called it threshold in this paper. When *θ *= 0, the ameliorated version of MI is traditional form of MI, so the ameliorated MI is an extend version of traditional MI. *b *is a real number and is greater than 1, it can be seen as penalty coefficient. Proper setting of the two parameters will make the positively associated symptoms keep their association invariant, while the negatively associated counterparts lessen their association, even turn to zero.

#### Correlative measure for continuous variables

Let us consider two continuous variables. Based on above definitions, now we want to reduce the correlative measure format for two continuous variables satisfied normal contribution [10].

Let two continuous variables *X*, *Y *satisfied normal contribution, their PDFs are

$$\begin{array}{c} f(x)=1/\left(\sqrt{2\pi }\sigma_{x}\right)\cdot \exp\left(-{\left(x-E_{x}\right)}^{2}/\left(2\sigma_{x}^{2}\right)\right)\\ f(y)=1/\left(\sqrt{2\pi }\sigma_{y}\right)\cdot \exp\left(-{\left(y-E_{y}\right)}^{2}/\left(2\sigma_{y}^{2}\right)\right) \end{array}$$

while -∞ <*x *< ∞, -∞ <*y *< ∞, *E*_*x*_, *E*_*y *_are mathematical expectations of *X*, *Y*, *σ*_*x*_, *σ*_*y *_are standard deviation of *X*, *Y*.

Joint probability density function of *X*, *Y *is expressed as

$$f(x,y)=\left[\frac{1}{2\pi \sigma_{x}\sigma_{y}\sqrt{1-\rho^{2}}}\right]\cdot \exp\left(\left[-\frac{1}{2(1-\rho^{2}}\right]\cdot \left[\left(\frac{{\left(x-E_{x}\right)}^{2}}{\sigma_{x}^{2}}-\frac{2\rho (x-E_{x})(x-E_{y})}{\sigma_{x}\sigma_{y}}+\frac{{\left(x-E_{y}\right)}^{2}}{\sigma_{y}^{2}}\right)\right]\right)$$

while *ρ *is correlation coefficient of *X*, *Y*.

Entropy of *X *is

$$\begin{array}{c} H(X)=-\int_{\text{-}\infty }^{\infty }f(x)\log(f(x))\text{d}x\\ =-\int_{\text{-}\infty }^{\infty }\frac{1}{\sqrt{2\pi }\sigma_{x}}\cdot \exp\left(-\frac{{\left(x-E_{x}\right)}^{2}}{2\sigma_{x}^{2}}\right)\log\left(\frac{1}{\sqrt{2\pi }\sigma_{x}}\cdot \exp\left(-\frac{{\left(x-E_{x}\right)}^{2}}{2\sigma_{x}^{2}}\right)\right)\text{d}x\\ =-\log\left(\frac{1}{\sqrt{2\pi }\sigma_{x}}\right)+1/2 \end{array}$$

In a similar way, entropy of *Y *is

$$H(Y)=-\log\left(\frac{1}{\sqrt{2\pi }\sigma_{y}}\right)+\frac{1}{2}$$

And then joint entropy of *X*, *Y *is

$$H(X,Y)=-\int_{\text{-}\infty }^{\infty }\int_{-\infty }^{\infty }f(x,y)\log\left(f(x,y)\right)\text{d}x\text{dy}$$

let *x *- (*μ*_*x*_/*σ*_*x*_) = *u*, *y *- (*μ*_*y*_/*σ*_*y*_) = *v*, then

$$\begin{array}{l} H(X,Y)\\ =-\int_{\text{-}\infty }^{\infty }\int_{-\infty }^{\infty }\left[\exp\left(\frac{-1}{2(1-\rho^{2})}\cdot \left(u^{2}-2\rho uv+v^{2}\right)\right)\right]\cdot \left[\log\left(\frac{1}{2\pi \sigma_{x}\sigma_{y}\sqrt{1-\rho^{2}}}\right)\right]\text{d}u\text{d}v+\\ \left[\frac{1}{2\pi {\left(1-\rho^{2}\right)}^{1/2}}\right]\cdot \int_{\text{-}\infty }^{\infty }\int_{-\infty }^{\infty }\left[\exp\left(\frac{-1}{2(1-\rho^{2})}\cdot \left(u^{2}-2\rho uv+v^{2}\right)\right)\right]\cdot \left[\frac{u^{2}-2\rho uv+v^{2}}{2(1-\rho^{2})}\right]\text{d}u\text{d}v\\ =-\log\left(\frac{1}{2\pi \sigma_{x}\sigma_{y}\sqrt{1-\rho^{2}}}\right)+1 \end{array}$$

So now we get correlative measure between *X*, *Y *is

(12) $$MI\left(X,Y\right)=H(X)+H(Y)-H(X,Y)=-\frac{\log(1-\rho^{2})}{2}$$

### Entropy partition method

Once association for each pair (every two variables) is acquired, we propose a self-organized algorithm to automatically discover the patterns. The algorithm can not only cluster, but also make some variables appear in some different patterns. In this section, we use three subsections to introduce the algorithm. In the first subsection, we introduce the concept of "Relative" set. Based on this, the pattern discovery algorithm is proposed in the second subsection. The last subsection is devoted to presenting an n-class association concept to back up the idea of the algorithm.

#### "Relative" set

For a specific variable *X*, a set, which is collected by means of gathering *N *variables whose associations with *X *are larger than others with regard to *X*, is attached to it and is denoted as *R*(*X*). Each variable in the set can be regarded as a "Relative" of *X *while other variables that do not belong to the set are considered as irrelative to *X*, so we name *R*(*X*) as "Relative" set of *X*. The "Relative" sets of all *k *variables can be denoted by a *k *× N matrix. Based on the matrix, the pattern discovery algorithm is proposed.

#### Algorithm steps

A pair (variable *X *and *Y*) is defined to be significantly associated if and only if *X *belongs to the "Relative" set of *Y *(*X *∈ *R*(*Y*)) and *vice versa *(*Y *∈ *R*(*X*)). It is convenient to extend this definition to a set with multiple variables. If and only if each pair of these variables is significantly associated, then we can call that the set is significant associated. A pattern is defined as a significantly associated set with maximal number of variables. All these kinds of sets constitute the hidden patterns in the data. Therefore, a pattern should follow three main criteria: (1) the number of variables within a set is no less than 2; (2) each pair of the variables belong to a set is significantly associated; and (3) any variable outside a set cannot make the set significantly associated. This means the number of variables within the set reaches maximum.

To discover all patterns hidden in the data, we propose the unsupervised algorithm, which can be implemented by three steps.

#### Step 1

Based on the *Q *× *N *matrix, all the significantly associated pairs are collected, denoted by a *M*_2 _× 2 matrix, where *M*_2 _represents the number of significantly associated pairs.

#### Step 2

Based on the *M*_2 _× 2 matrix, collecting all the significantly associated three variables, denoted by a *M*_3 _× 3 matrix, where *M*_3 _represents the number of significantly associated three variables. Similarly, if there exist significantly associated multiple variables, the corresponding result is denoted by *M*_*m *_× *m*, where *M*_*m *_represents the number of significantly associated multiple variables and *m *stands for the number of variables. Obviously *m *≤ *N*, where *N *represents the number of relative variables. Since *N *is bounded, the algorithm can converge.

#### Step 3

Finding the maximal *m*. Matrix *M*_*m *_× *m *have *M*_*m *_patterns with *m *variables. A set that contains *m*-1 variables is certainly not a pattern since it does not fulfill the third criterion of a pattern. All these kinds of sets are removed from the matrix *M*_*m*-1 _× (*m *- 1), the rest are certainly patterns with *m *- 1 variables. Similarly, all the patterns can be discovered.

#### N-class association

From pattern discovery method introduced above, we know that it needs to compute $C_{n}^{2}$ times between every two symptoms of *n *symptoms and this number will rise into $C_{n}^{3}$ if for every three symptoms of *n *symptoms. Generally speaking, there are many cases that several (such as 5 or 6) symptoms combine together to describe syndrome in TCM theory. Therefore, based on the unsupervised character of the data, the number of symptoms to be computed should be 2~3 multiples of actual 5~6, namely 10~18. With regard to the TCM data of this paper, the computation times number reaches $C_{72}^{10}\sim C_{72}^{18}$, namely about 10^16 ^magnitude, which doesn't match any general computer's capacity currently. Because of the above-mentioned reasons, we introduce the concept of *n*-class association. Formally speaking, for *n *variables, if arbitrary *n *- 1 variables of the *n *variables are considered as close association, then we can call these *n *variables are of *n*-class association. Specially, when *n *= 2, it is just about the association between two variables.

Base on the concept of *n*-class association, when turning to judge *n *variables are associated or not, we need only to judge whether arbitrary *n *-1 variables of *n *variables are associated. It means that, theoretically we just need to implement the computing of the association between two variables, which significantly decreases the computation complexity so that the pattern discovery algorithm could be applied into large-scale data. In fact, thanks to mathematical induction, the *n*-class association concept is easy to understand on the mathematics.

For *n *= 2, proof of this proposition is obvious. Supposed that *n *variables are close associated, and our purpose is to prove that when an (*n *+ 1)-th variable is under close association with other *n *variables, the whole (*n *+ 1) variables are considered close associated.

We know that $MI(X_{1},X_{2},\cdots ,X_{n})=\sum_{i=1}^{n}H(X_{i})-H\left(\sum_{i=1}^{n}X_{i}\right)$, then

$$\begin{array}{l} MI\left(X_{1},X_{2},\cdots ,X_{n},X_{n+1}\right)\\ =\sum_{i=1}^{n}H\left(X_{i}\right)+H\left(X_{n+1}\right)-H\left(\sum_{i=1}^{n+1}X_{i}\right)\\ =\sum_{i=1}^{n}H\left(X_{i}\right)+H\left(X_{n+1}\right)-H\left(\sum_{i=1}^{n}X_{i},X_{n+1}\right)\\ =\sum_{i=1}^{n}H\left(X_{i}\right)+H\left(X_{n+1}\right)-\left[H\left(\sum_{i=1}^{n}X_{i}\right)+H\left(X_{n+1}\right)-MI\left(\sum_{i=1}^{n}X_{i},X_{n+1}\right)\right]\\ =\left[\sum_{i=1}^{n}H\left(X_{i}\right)-H\left(\sum_{i=1}^{n}X_{i}\right)\right]+MI\left(\sum_{i=1}^{n}X_{i},X_{n+1}\right)\\ =MI\left(X_{1},X_{2},\cdots ,X_{n}\right)+MI\left(\sum_{i=1}^{n}X_{i},X_{n+1}\right) \end{array}$$

It tells us that the correlative measure among *X*_1_, *X*_2_,⋯, *X*_*n*_, *X*_*n*+1 _is composed of the association measure among *X*_1_, *X*_2_,⋯, *X*_*n *_and measure between two subset *X*_1_, *X*_2_,⋯, *X*_*n *_and *X*_*n*+1_. It means that if an *n *+ 1-th variable is under close association with other *n *variables, these *n *+ 1 variables are considered as close association. The proposal of *n*-class association concept extensively decreases the computational complexity of pattern discovery algorithm.

### Validation method

#### Algorithm steps

To validate the algorithm and illustrate the reason of choosing parameters as described above, we must take the objective data for the unsupervised data into account.

In the supervised learning situation, the validation of the algorithm is performed by estimating three measures: sensitivity, specificity and accuracy, of the classification results [11]. However, under unsupervised background here, validation of the algorithm must be done in a slightly different way. We summarize it in following three steps.

#### Step 1

For each pattern *S*, we return it to the unsupervised data, if all variables of the pattern simultaneously appear (their values are non-zero) on a patient, then serial number of the patient is recorded. We collect all these serial numbers, enumerate the total numbers of them, record the number as *L*_*S*_. All the serial numbers are stored in a vector with *L*_*S *_dimensions denoted as ${\vec{V}}_{S}$.

#### Step 2

Tracking the vector ${\vec{V}}_{S}$ to the syndrome data, we get *L*_*S *_vectors with 9 dimensions. Each dimension encodes a syndrome uniformly. The *L*_*S *_vectors are added one by one to generate a new vector $\vec{W_{S}}$ = *w*^*s*^_*i*_, *i *= 1, 2,..., 8, 9), where *i *represents the *i-th *syndrome, *w*^*s*^_*i *_denotes that there are *w*^*s*^_*i *_patients are diagnosed as the syndrome in the whole *L*_*S *_patients. Obviously, we have *w*^*s*^_*i *_≤have enough NEI data of 400 Wistar *L*_*s*_. It is easy to find the maximal number, denoted as $w_{i_{\max}}^{S}$, in the vector $\vec{W_{S}}$. We record the $w_{i_{\max}}^{S}$ and the corresponding syndrome *i*_*max*_.

#### Step 3

We define the sensitivity *T*_*s *_of the pattern *S *as $T_{S}=\frac{w_{i_{\max}}^{S}}{L_{S}}$. The sensitivity of the algorithm, denoted as *T*, can be calculated by summing up sensitivities of all patterns and then averaging. i.e. $T=\frac{1}{P}\sum_{S=1}^{P}T_{S}$, where *P *denotes the number of patterns.

#### Relation between sensitivity of algorithm and threshold

Given a clinical data, the number of patterns, denoted as *P *above, generated by the algorithm is only determined by the number of "relative" N and threshold *θ*, i.e., *P *= *f*(*N*, *θ*). We now reconsider the form of the sensitivity of the algorithm *T*:

(13) $$\begin{array}{l} T=\frac{1}{P}\sum_{S=1}^{P}T_{S}\\ =\frac{1}{f(N,\theta )}\sum_{S=1}^{f(N,\theta )}T_{S}\\ =\frac{1}{f(N,\theta )}\sum_{S=1}^{f(N,\theta )}\frac{w_{i_{\max},S}}{L_{S}} \end{array}$$

Where $w_{i_{\max}}^{S}$ and *L*_*S *_are constant for a given data, so *T *is determined by N and *θ*. For clinics, a pattern with 3 or 4 variables is optimal to be diagnosed as what syndrome, so N is chosen to be not less than 4.

## Results and discussion

### Discrete data example

#### Data collection

Syndrome is diagnosed according to symptom combinations. As shown in Table 1, we choose 72 symptoms that are closely related to CRF. The pulse information of every patient was not included for its bad consistency during the process of survey. In the survey, the data set was recruited from six clinical centers located in six provinces from the same demographic area and at the same time from October 2005 to March 2006, where a total of 601 patients who suffer from CRF were surveyed.

**Table 1:** The name of each variable and its frequency. The most is Hypodynamia, the least is Anuria. The total patients number is 601.

**No**	**Name**	**Frequency**	**No.**	**Name**	**Frequency**
**1**	Fear of cold	69.6%	**37**	Abdominal distention	26.0%
**2**	Feverishness in palms and soles	34.3%	**38**	Abdominal pain	9.8%
**3**	Spontaneous perspiration	26.3%	**39**	Lumbago	48.1%
**4**	Night sweat	17.5%	**40**	Soreness and weakness of waist and knees	71.2%
**5**	Spiritlessness	66.9%	**41**	Cold waist and knee	28.6%
**6**	**Hypodynamia**	**87.0%**	**42**	Unwarm of hands and feets	33.1%
**7**	Disinclination to say	41.43%	**43**	Tetany	33.6%
**8**	Dysphoria	53.1%	**44**	Numbness of limbs	25.6%
**9**	Deprementia	35.9%	**45**	Pain of limbs	13.6%
**10**	Somatic heaviness	53.2%	**46**	Anorexia	55.4%
**11**	Amnesia	59.4%	**47**	Sticky mouth	37.6%
**12**	Asteatosis cutis	61.1%	**48**	Tastelessness	41.1%
**13**	Itch of skin	45.4%	**49**	Bitter taste of mouth	30.4%
**14**	Petechia on skin	0.08%	**50**	Salty taste of mouth	5.8%
**15**	Scaly skin	17.0%	**51**	Dry mouth	63.7%
**16**	Eclipse on complexion	44.1%	**52**	Halitosis	29.1%
**17**	Headache	34.4%	**53**	Thirst without desire to drink	16.3%
**18**	Lightheadedness	50.7%	**54**	Thirst and desire to drink	49.1%
**19**	Distention of head	29.6%	**55**	Insomnia	55.2%
**20**	Cerebaria	30.3%	**56**	Dreaminess	55.2%
**21**	Yellow complexion	57.7%	**57**	Drowsiness	19.3%
**22**	Pale complexion	11.3%	**58**	Loose stool	20.3%
**23**	Red complexion	4.5%	**59**	Constipation	32.8%
**24**	Greenish complexion	6.3%	**60**	Diarrhea	10.0%
**25**	Dry eye	47.4%	**61**	Unwell stool	23.5%
**26**	Dizziness	33.3%	**62**	Clear urine in large amounts	24.0%
**27**	Bombus	46.8%	**63**	Deep-colored urine	48.6%
**28**	Dry pharynx	65.4%	**64**	Frequent micturition	30.1%
**29**	Cardiopalmus	48.6%	**65**	Frequent nocturia	66.9%
**30**	Chest distress	46.3%	**66**	Oliguria	5.8%
**31**	Short breath	45.8%	**67**	**Anuria**	**0.8%**
**32**	Hypochondriac distension	15.3%	**68**	Puffiness of face	37.3%
**33**	Hypochondriac pain	7.5%	**69**	Puffiness of upper limb	9.8%
**34**	Nausea	48.1%	**70**	Puffiness of lower limb	45.4%
**35**	Emesia	25.5%	**71**	Pleural fluid	4.7%
**36**	Gastric cavity	41.4%	**72**	Abdominal dropsy	4.7%

The case must strictly meet four conditions to be included within the data: (1) based on the diagnosis criterion of CRF and the state of illness to be classified under stages 3, 4 and 5 [12]; (2) no dialysis therapy for all patients for a month before the survey; (3) patients of ages between 18 and 65 years; and (4) patients must agree to sign the informed consent. Additionally, there is also three exclusion criteria information contained in the survey.

(1) Besides chronic kidney function failure, a patient also suffers from inter-current diseases such as serious respiratory, cardiovascular, cerebrovascular, and digestive blood system diseases.

(2) Women who are in gestation or lactation will be excluded.

(3) A patient with symptoms produced by drug therapy.

Every case is with 72 symptoms, together with the basic information of each subject. The frequencies of 72 symptoms are listed in Table 1, each variable (symptom) has four categories, i.e. none, light, middle, severe, represented by 0, 1, 2, 3, respectively. The latter three categories of each variable mean that the symptom has appeared and then separated into light, middle, severe by clinical doctors, who are strictly and uniformly trained to reach a high consistency.

#### Diagnostic data

CRF patients recruited here were clinically diagnosed by TCM physicians to receive herbal treatment. We collected this diagnostic data (also called syndrome data) to validate the unsupervised pattern discovery algorithm. The data is composed of nine syndromes. Name and frequency of syndromes are shown in Table 2 in a frequency-descending way. The data is represented by a matrix, row represents an observation, and column represents a syndrome. If a patient is diagnosed as one of nine syndromes, the corresponding column of the matrix is denoted as 1, otherwise the column is denoted as 0. Generally speaking, if a CRF patient is diagnosed having two syndromes or above, the corresponding columns are all denoted as 1.

**Table 2:** The basic information of syndrome data. Each syndrome is assigned a Greek symbol. The total patients number is 601.

**Syndrome**	**Frequency**	**Syndrome**	**Frequency**
□: Deficiency of Qi	480	□: Qi stagnation	69
□: Deficiency of Yin	347	□: Endogenous heat	52
□: Fluid-dampness	321	□: Yang-invigoration	4
□: Deficiency of Yang	299	□: Endogenous cold	3
□: Blood stasis	117		

#### Parameter setting

The algorithm has three parameters to be adjusted. First, three or four symptoms usually constitute a syndrome. On the other hand, in clinical application, too many symptoms (like eight symptoms) may confound the TCM physicians and lead to complex result, thus in our model we set the number of "Relatives" of variables, denoted as N, 5. Second, we set the threshold as: *θ *= 40/601 = 6.67%. This parameter choice involves validation of the algorithm steps section. Third, penalty coefficient b is set as 2, which will separate positive and negative associations in our model.

#### Partition result and discussion

As depicted in Part A of Table 3, one pattern have four symptoms, the other 15 patterns are comprised of three symptoms. The 35 patterns with two symptoms are not listed here for their minor contribution to clinics, since it is very hard to diagnose two symptoms as a syndrome in clinics. Here, a pattern including more than two symptoms is called a clinically effective pattern.

**Table 3:** Patterns discovered automatically by the algorithm

	**Part A: Unsupervised partition**		**Treatment**	**Part B: Supervised validation**		
			
**No**	**Patterns***S*	**Syndrome**		**Cases**	**max number**	**Sensitivity**
1	Somatic heaviness; Cardiopalmus; Chest distress; Short breath	Deficiency of Qi	Shenling baizhu powder	133	130(1)	97.74%
2	Fear of cold; Cold waist and knee; Unwarm of hands and feets	Deficiency of Yang	Jisheng shenqi pills	86	86(7)	100%
3	Feverishness in palms and soles; Spontaneous perspiration; Night sweat	Deficiency of Yi	Danggui liuhuang decoction	33	33(5)	100%
4	Spiritlessness; Hypodynamia; Disinclination to say	Deficiency of Qi	Sijunzi decoction	220	217(1)	98.64%
5	Spiritlessness; Disinclination to say; Deprementia	Deficiency of Qi	Sijunzi decoction	142	140(1)	98.59%
6	Hypodynamia; Somatic heaviness; Chest distress	Dampness and Deficiency of Qi	Pingwei powder	188	180(1)	95.74%
7	Disinclination to say; Anorexia; Tastelessness	Deficiency of Qi	Sijunzi decoction	121	117(1)	96.69%
8	Asteatosis cutis; Itch of skin; Scaly skin;	Blood stasis	Danggui yinzi	69	61(6)	88.41%
9	Itch of skin; Scaly skin; Numbness of limbs	Blood stasis	Danggui yinzi	37	32(6)	86.49%
10	Itch of skin; Unwarm of hands and feets; Numbness of limbs	Deficiency of Yang	Danggui yinzi	57	53(7)	92.98%
11	Dry pharynx;Dry mouth;Thirst and desire to drink	Endogenous heat	Wuling powder	198	198(5)	100%
12	Chest distress;Short breath;Hypochondriac distension	Qi stagnation	Wumo yinzi	59	55(2)	93.22%
13	Hypochondriac distension;Gastric cavity; Abdominal distention	Qi stagnation	Wumo yinzi	47	47(2)	100%
14	Nausea; emesia;Anorexia	Dampness-heat	Shenling baizhu powder	115	110(1)	95.65%
15	emesia;Anorexia; Tastelessness	Dampness-heat	Shenling baizhu powder	72	70(1)	97.22%
16	Puffiness of face;Puffiness of upper limb;puffiness of lower limb;	Fluid-dampness	Wupi powder	43	43(3)	100%

Part A shows the 16 patterns and the corresponding syndrome name in the unsupervised partition process. Each pattern can be diagnosed as a syndrome. Different treatments aim to different syndromes. Part B shows the cases, max number and the sensitivity in the supervised validation process.

**Figure 1:**
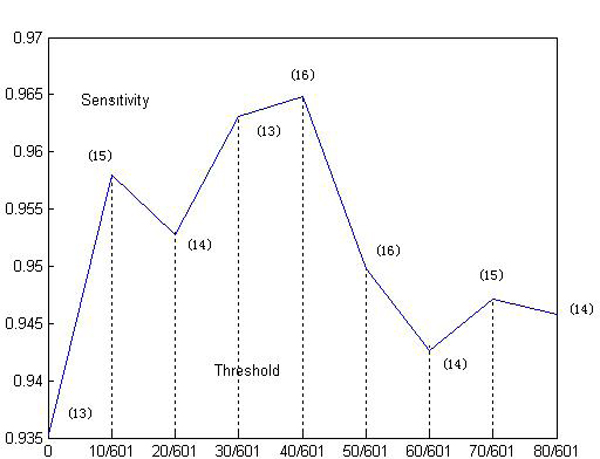
**Sensitivity of the algorithm versus threshold of the algorithm**. The number in the bracket around each point denotes the number of effective patterns. The maximal sensitivity is 0.9648

**Figure 2:**
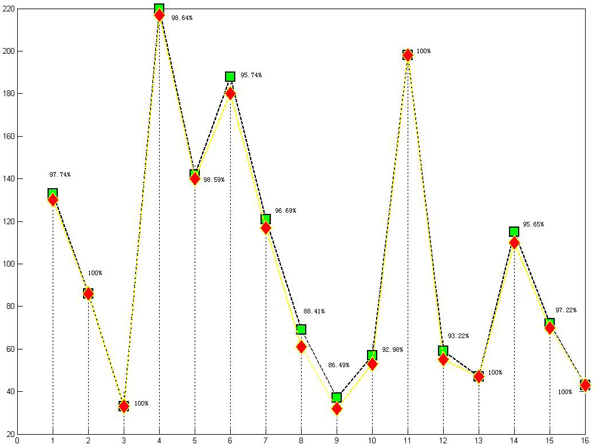
**X-axis is pattern, Y-axis is corresponding number of cases in the data**. For green square point, the data is symptoms data, while for red diamond point, the data is syndrome data. The percentage around each point indicates the sensitivity of the corresponding pattern. The average of them is sensitivity of the algorithm – 96.48%.

Here, we investigate the relation between the sensitivity of the algorithm *T *and the threshold *θ*. As depicted in Figure 1, sensitivity of the algorithm is varied in different threshold. The optimal threshold is 40/601 and the corresponding largest sensitivity is 0.9648, which is better than any reported literature till now. When the threshold is 0, the form of MI is turned into the traditional MI. From Figure 1 we can easily see that the ameliorated version of MI is better than traditional MI since sensitivities of the algorithm in the situation of threshold *θ *> 0 are larger than the counterpart in *θ *= 0. The larger the sensitivity of the algorithm, the corresponding result more accords with the clinics. The number in the bracket around each point means the number of effective patterns discovered in the corresponding threshold setting. As depicted in Figure. 2, each pattern's distribution in patient data is and its corresponding sensitivity are showed. The pattern's sensitivity is defined in Step 3 of validation method's algorithm steps.

### Continuous data example

#### Data collection

For continuous variables, we use the neuro-endocrine-immune data collected from 400 Wistar rats. In 1977 Besedovsky proposed "immune-neuro-endocrine network" theory [13], and this theory has broadly studied and rapidly become one important theory hotspot in medicine and biology field. More and more evidences indicated that there are not only one big loop among these three systems but also direct and bidirectional interaction between each other [14-16].

We have enough NEI data of 400 Wistar rats collected by the following ways and means:

• This research focuses on the high risk factors and pathogenesis of vascular disease and takes vascular endothelial function as breakthrough point.

• This research divides 400 Wistar rats to 6 groups by using the randomized single blind method: normal group; basic model group; composite model group; ginseng intervention group; double-ginseng intervention group, compound powder intervention group.

• The rats of basic model group are fed with hyperhomocysteinemic of fixed ration to come into being endothelial dysfunction.

• Base on basic model group, the rats of composite model group are forced to swim with fixed load at fixed time.

• Base on composite model group, the rats of ginseng intervention group are treated with ginseng of fixed ration.

• Base on composite model group, the rats of double-ginseng intervention group are treated with double-ginseng of fixed ration.

• Base on composite model group, the rats of compound powder intervention group are treated with compound powder of fixed ration.

• This research determines 5 vascular endothelial function related parameters and 21 NEI network related parameters. Each rat can only be measured several (usually 4 at most) parameters because of its limited blood volume. All the parameters are showed in Table 4, 5.

**Table 4:** Vascular endothelial function related parameters

**Parameter No.**	**Parameter symbol**	**Parameter name**
1	AngII	angiotensin 2
2	ET	endothelin
3	TXA2	thromboxane A2
4	PGI2	prostacyclin I2
5	NO	nitric monoxide

**Table 5:** NEI network related parameters

**Parameter No.**	**Parameter symbol**	**Parameter name**
1	PRA	plasma renin activity
2	AngII	angiotensin 2
3	ALD	aldosterone
4	GC	sugar corticosteroid
5	ACTH	adrenocorticotropic hormone
6	CRH	cortex releasing hormone
7	NE	norepinephrine
8	IgG	immunoglobulin G
9	IgA	immunoglobulin A
10	IgM	immunoglobulin M
11	C3	complement 3
12	C4	complement 4
13	CD4	cluster of differentiation 4
14	CD8	cluster of differentiation 8
15	IL-1	interleukin-1
16	IL-2	interleukin-2
17	IL-6	interleukin-6
18	IL-10	interleukin-10
19	hsCRP	high-sensitivity C-reactive protein
20	INF-*γ*	interferon *γ*
21	TNF-*α*	tumor necrosis factor *α*

#### Partition result and discussion

This research focus on finding whether there are some rules between vascular endothelial function related parameters and NEI network related parameters. According to the entropy partition algorithm, we take the NEI network related parameters as initial set *X*, and take the vascular endothelial function related parameters as object set *Y*. Both *X *and *Y *are prescribed as normal contribution. Corresponding parameters *σ*, *μ *can be estimated with Bayesian method [10]. After determining the density function of *X*, *Y*, we can calculate their correlative measure according to (12). Then we can take entropy partition to this data. According to *N *- *class *association, we can get each parameter's Relative set, thus final output set *S*.

The partition result is showed in Table 6. We can find most relevant NEI network related parameters of each vascular endothelial function related parameter. The parameter's relativity follows descending from top to down.

**Table 6:** Entropy partition result of rats data.

**Pattern**	**A**	**B**	**C**	**D**	**E**
	AngII	ET	TXA2	PGI2	NO

↓	ET	CRH	INF-*γ*	ALD	ALD
	IL-1	ACTH			
		GC			
		ALD			

Here shows the five patterns partitioned from rats data. Every pattern consisted of two parts: the first part from the vascular endothelial function related parameter showed in the second row, and the second part from NEI network related parameters showed in the rest rows. The arrowhead shows the correlative degree with the vascular endothelial function related parameter in every patteren.

**Figure 3:**
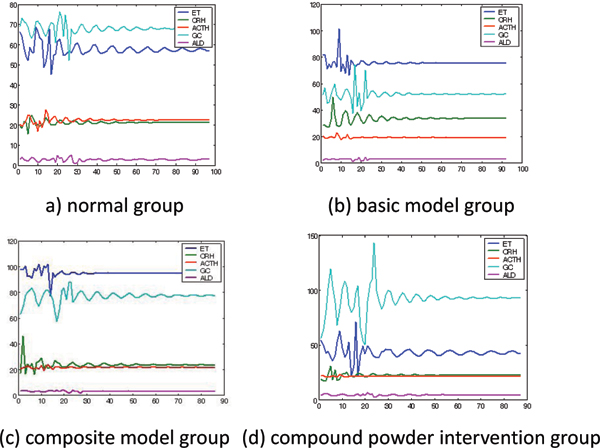
Most relevant NEI network related parameters of ET (pattern A).

We take pattern A in Table 6 for example. Figure 3 describes the rules consisting in the above-mentioned two parameters set. These figures show most relevant NEI related parameters to each vascular endothelial function related parameter. Through this way, we can find some interesting phenomena and rules.

The abnormity and disorder in NEI network will inevitably lead to some corresponding endothelial dysfunction. Take the composite model group for example, there will be some subsystems consisting of some endothelial function related parameters and some NEI network related parameters and they will exist in some laws during the remaining time.

When intervened by ginseng, double-ginseng, compound powder respectively, these rules in these subsystems receive varying degree of change. And after intervened by compound powder, these rules almost disappear and the trend looks like to be consistent with normal group.

This shows that the hyperhomocysteinemic feed in basic model group and load swimming in composite model group are just the particular causation of above-mentioned rules.

## Conclusion

In this paper, we presented an unsupervised partition method based on association delineated by revised mutual information. A revised version of mutual information is developed to discriminate positive association and negative counterpart. Based on our model, unsupervised pattern discovery algorithm is proposed to allocate significantly associated symptoms into several patterns. The algorithm not only can cluster, but also can make some symptoms appear in different patterns, which are consistent with TCM diagnoses. By using the syndrome data, the unsupervised algorithm was validated and the sensitivity of algorithm performance measure was defined to evaluate the patterns discovered. The algorithm reaches a maximal sensitivity with 96.48%, which means that the CRF data is of good quality. Furthermore, the results shows that, under proper parameters setting, the algorithm successfully discovered 16 patterns in CRF patients and each of the patterns can be automatically diagnosed as syndrome, which is completely in accordance with the corresponding results diagnosed by TCM physicians. We also apply this method in NEI data collected by400 Wistar rats, and the result shows some corresponding rules of vascular endothelial function related parameters and NEI network related parameters. The study in this paper provides an improved solution for syndrome classification in patient and rat data and its results can contribute significantly to the TCM practice.

## List of abbreviations used

TCM: Traditional Chinese Medicine; NEI: Neuro-Endocrine-Immune; MI: Mutual Information; CRF: Chronic Renal Failure

## Competing interests

The authors declare that they have no competing interests.

## Authors' contributions

JC developed the method and performed the results validation. GCX conceived of the study, and participated in its design and coordination and helped to draft the manuscript. All authors read and approved the final manuscript.
